# P-877. Effectiveness of Oral Step-down Antibiotic Therapy in Uncomplicated *Enterococcus faecalis* Bloodstream Infection

**DOI:** 10.1093/ofid/ofae631.1068

**Published:** 2025-01-29

**Authors:** Sarah Al Mansi, Margaret Pokalsky, Katherine Turnley, Andrew Freeman, P Brandon Bookstaver, Joseph Kohn, Hana R Winders, Sarah Withers, Majdi N Al-Hasan

**Affiliations:** University of South Carolina School of Medicine - Prisma Health Midlands, Columbia, SC; University of South Carolina School of Medicine, Columbia, South Carolina; University of South Carolina School of Medicine, Columbia, South Carolina; University of South Carolina School of Medicine, Columbia, South Carolina; Prisma Health Richland - University of South Carolina, Columbia, South Carolina; Prisma Health Midlands, Columbia, South Carolina; Prisma Health Richland, Columbia, South Carolina; Prisma Health, Greenville, South Carolina; University of South Carolina School of Medicine, Columbia, South Carolina

## Abstract

**Background:**

The transition from intravenous (IV) to oral antibiotic therapy is a common strategy in the management of uncomplicated bloodstream infection (BSI). However, the role of oral step-down therapy in uncomplicated *Enterococcus faecalis* BSI has not been defined. This retrospective cohort study examines the effectiveness of oral step-down antibiotics compared to standard IV therapy in uncomplicated *E. faecalis* BSI.

Risk factors for treatment failure in uncomplicated Enterococcus faecalis bloodstream infection (BSI)

**Figure:**
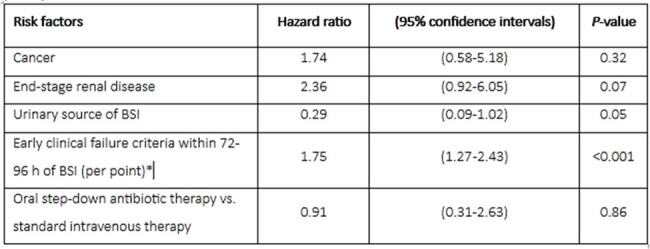

*Early clinical failure criteria include systolic blood pressure <100 mmHg or vasopressor use, heart rate >100 beats/minute, respiratory rate ≥ 22 breaths/minute or mechanical ventilation, altered mental status, white blood cell count >12,000/mm3.

**Methods:**

Adults with uncomplicated *E. faecalis* BSI admitted to 10 Prisma Health hospitals in South Carolina from January 2021 to June 2023 were evaluated. Clinically insignificant positive blood cultures, polymicrobial, recurrent, persistent, and complicated BSI were excluded. Deaths within 7 days were excluded to mitigate immortal time bias. Multivariate Cox proportional hazards regression examined the risk of treatment failure (all-cause mortality or recurrence) within 90-day. Wilcoxon rank sum test compared hospital length of stay (HLOS) between the two groups.

**Results:**

Of 476 screened patients, 131 with uncomplicated *E. faecalis* BSI were included in the analysis. The median age was 70 years, 84 (64%) were men, and 46 (35%) had a urinary source of infection. Eighty-seven patients (66%) received standard IV therapy and 44 (34%) were transitioned to oral step-down therapy. Median antibiotic treatment durations were 15 and 14 days, respectively. Patients in the oral step-down group received 6 days of IV antibiotics followed by 8 days of oral therapy, most commonly oral aminopenicillins (33/44; 75%). In the multivariate Cox model, oral step-down therapy was not associated with increased risk of treatment failure compared to standard IV therapy (hazard ratio 0.91, 95% confidence intervals 0.31-2.63; Table). HLOS was 7 days in the oral step-down group and 11 days in the standard IV group (p< 0.001).

**Conclusion:**

Transitioning patients with uncomplicated *E. faecalis* BSI from IV to oral step-down antibiotic therapy appears to be a promising strategy with shorter HLOS and no significant increase in the risk of treatment failure. These results should be confirmed in larger multicenter studies prior to routine implementation in clinical practice.

**Disclosures:**

**All Authors**: No reported disclosures

